# PBS-Based Green Copolymer as an Efficient Compatibilizer in Thermoplastic Inedible Wheat Flour/Poly(butylene succinate) Blends

**DOI:** 10.1021/acs.biomac.0c00701

**Published:** 2020-06-30

**Authors:** Michelina Soccio, Franco Dominici, Silvia Quattrosoldi, Francesca Luzi, Andrea Munari, Luigi Torre, Nadia Lotti, Debora Puglia

**Affiliations:** † Civil, Chemical, Environmental and Materials Engineering Department, University of Bologna, Via Terracini 28, 40131 Bologna, Italy; ‡ Civil and Environmental Engineering Department, University of Perugia, Strada di Pentima 4, 05100 Terni, Italy

## Abstract

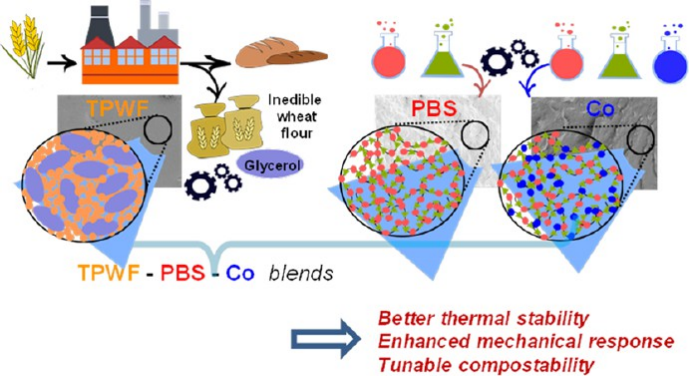

Considering the current context of research aiming at proposing new bioplastics with low costs and properties similar to fossil-based commodities currently on the market, in the present work, a hybrid blend containing a prevalent amount of cheap inedible cereal flour (70 wt %) and poly(butylene succinate) (PBS) (30 wt %) has been prepared by a simple, eco-friendly, and low-cost processing methodology. In order to improve the interfacial tension and enhance the adhesion between the different phases at the solid state, with consequent improvement in microstructure uniformity and in material mechanical and adhesive performance, the PBS fraction in the blend was replaced with variable amounts (0–25 wt %) of PBS-based green copolymer, which exerted the function of a compatibilizer. The copolymer is characterized by an ad hoc chemical structure, containing six-carbon aliphatic rings, also present in the flour starch structure. The two synthetic polyesters obtained through two-stage melt polycondensation have been deeply characterized from the molecular, thermal, and mechanical points of view. Copolymerization deeply impacts the polymer final properties, the crystallizing ability, and stiffness of the PBS homopolymer being reduced. Also, the prepared ternary blends were deeply investigated in terms of microstructure, thermal, and mechanical properties. Lastly, both pure blend components and ternary blends were subjected to disintegration experiments under composting conditions. The results obtained proved how effective was the compatibilizer action of the copolymer, as evidenced by the investigation conducted on morphology and mechanical properties. Specifically, the mixtures with 15 and 20 wt % Co appeared to be characterized by the best mechanical performance, showing a progressive increase of deformation while preserving good values of elastic modulus and stress. The disintegration rate in compost was found to be higher for the lower amount of copolymer in the ternary blend. However, after 90 days of incubation, the blend richest in copolymer content lost 62% of weight.

## 1 Introduction

Governments, citizens, and companies currently recognize that plastics have a negative impact besides the great benefits they bring to our lives. The types of plastics we use and dispose as waste have serious implications for human health and the environment. The transition to plastics meeting our needs, while being harmless to both human and animal health and to the environment, has become a mandatory and urgent problem. Science, industry, and public policies are working to encourage the introduction of eco-friendly materials. Among these plastics, thermoplastic starch (TPS), obtained when the ordered granular structure of starch is disrupted by heating with a plasticizer or gelatinization agent,^1^ could represent a solution. TPS has gotten increasing importance in recent years as it is economically viable, classified as biodegradable or compostable, and can be obtained from a range of native sources, such as wheat, rice, corn, potato, pea, and cassava.^2−4^ However, pure TPS has several drawbacks, such as brittleness, too high degradation rate in environmental conditions, and mechanical properties that are very sensitive to moisture content. Its blending with synthetic low-molecular-weight molecules and polymers represents an effective strategy to overcome these problems, being blending a simple, rapid, and cheap method to achieve a proper combination of properties not generally obtainable with a single polymeric material.^4−6^ To improve its processability and mechanical properties, TPS, obtained by treating starch with glycerol, has been blended with traditional fossil-based polyethylene (PE),^7,8^ polypropylene (PP),^9^ and polyamide (PA).^10,11^

Several blends of TPS with various completely biodegradable polymers have been also developed to achieve a totally biodegradable material.^12,13^ Among these biodegradable polymers, noteworthy is poly(butylene succinate) (PBS), obtainable from 100% renewable resources, characterized by a wide workability window and mechanical response very similar to the LDPE commodity polymer.

Most polymer blends are immiscible and need to be compatibilized. Compatibilization, in most cases, is realized by addition of a compatibilizer, being necessary to guarantee (i) optimization of the interfacial tension, (ii) stabilization of the morphology against high stresses during forming, and (iii) enhanced adhesion between the phases in the solid state, with consequent improvement in microstructure uniformity but also in material performance, specifically mechanical and adhesive properties. The combination of starch with PBS has been widely studied, with the response of both gelatinized and untreated starch mixed with PBS confirming their limited miscibility if not properly plasticized and chemically modified.^14−16^ Zeng et al. reported that PBS and plasticized starch can be blended in the presence of reactive PBS phases (NCO-terminated), which produced blends characterized by improved tensile strength, remarkably superior to thermoplastic starch (TPS), and at the same time enhanced hydrophobicity with consequent reduction of water absorption. The improvement obtained was higher with higher content of reactive poly(butylene succinate) (RPBS).^17^ Another interesting in situ compatibilization approach has been considered by Suchao-In et al.^18^ In this case, PBS was grafted on starch through a single step process to obtain starch-*g*-PBS. The possibility of using maleated PBS was taken into account by different authors.^19,20^ The published papers showed that the addition of RPBS to TPS/PBS blends guarantees a significant improvement of the mechanical response in terms of strength and elongation at break due to the formation of smaller phase domains, better distributed, especially in higher content of compatibilizer.

The effect of plant oil addition in a quite low amount (0.5 wt %) was also tested,^21^ and also, in this case, the TPS/PBS films showed significantly improved mechanical properties. Recently, Zhang et al. demonstrated that mixtures of starch, glycerol, and tartaric acid (TPS-TA) prepared by reactive extrusion were very effective in altering positively the impact strength of PBS/TPS-TA, with TA inducing “sea-island” morphology.^22^ If several groups have studied the effect on the final material properties of different flours, such as rice^23^ and corn,^24,25^ few groups have been dealing with the effect of starch origin, meaning amylose or amylopectin content, on blend miscibility. Li et al.^26^ and Ayu et al.^25^ used waxy (0% amylose) and normal (26% amylose) corn starches to prepare blends of poly(butylene succinate) and thermoplastic starch (WTPS and NTPS, respectively). The results described in these papers indicated that the plasticization and processing of waxy corn starch were easier than those of normal corn starch and the combination of PBS and WTPS produced some excellent performances, such as good processability, superior mechanical properties, and higher water resistance.

To the best of the authors’ knowledge, there are no reports on plasticized flours with significant protein contents, with the exception of a paper from Ku-marsilla and Verbeek,^27^ who analyzed blends of PBS and thermoplastic protein set at 50 wt % in the presence or absence of compatibilizers (poly(methylene diphenyl diisocyanate) (pMDI) and poly(2-ethyl-2-oxazoline)) at variable loadings, dissolved in water prior to extrusion, with methylene diphenyl diisocyanate showing less water absorption compared to not compatibilized blends.

In our work, we attempted, for the first time, the realization of PBS-based blends containing high content of plasticized wheat flour (TPWF), characterized by alveographic parameters typical of a soft grain cultivar, which is able to give more deformable thermoplastic films.

It has to be emphasized that, in the present paper, a cheap inedible cereal flour (as in the case of wheat grains and flours contaminated by fungi or infested by insects and rodents, which undergo sanitary restrictions) has been employed in place of chemically refined starches to prepare blends with PBS, whose cost is still relatively high, by adopting a processing methodology that should avoid the environmental high costs of reactive extrusion with a maleated fraction as the compatibilizer agent. According to this, the blends were realized by considering a prevalent content of plasticized wheat flour (70 wt %) with a moderate amount of polymer matrix (30 wt %). To favor the TPWF/PBS compatibilization, both from the chemical and physical points of view, variable fractions (0–25 wt %) of an ad hoc synthetized PBS-based copolymer, containing Pripol 1009 moieties, have been employed. In this copolymeric system, as previously reported by some of us,^28^ the succinic acid subunit was partially replaced with the Pripol 1009 subunit, the last containing an aliphatic six-carbon atom ring connected to the −COOR groups through PE-like segments and presenting quite long side branches. The insertion of the aliphatic ring in the polymer matrix, also present in the flour starch structure, should enhance the TPWF/PBS chemical compatibility. Moreover, the PE-like sequences present both in the main chain and as side branches increase the macromolecular mobility and, as a consequence, decrease the elastic modulus with respect to the PBS homopolymer,^28^ favoring also the physical compatibility between the TPWF matrix and polymer component. Besides, the higher thermal stability and the lower melting temperature of the P(BS-*co*-Pripol) system^28^ allows limiting undesired thermal degradation of the final material. Finally, the compatibilizer also permits tuning the composting rate^28^ according to the final application envisioned for the composites.

## 2 Experimental Part

### 2.1 Materials

Mantegna wheat flour was selected after a preliminary screening among a total of 30 provided by ASSAM Marche (Jesi, Ancona Province). Its soft grains were ground by a laboratory mill with a standard procedure and machinery adjustment. The protein content was 12.6%, while its alveographic parameters were measured (*P*/*L* = 0.21, *W* = 63). Selected flour was plasticized with 23%, w/w of glycerol, as reported elsewhere.^29,30^

Butanediol (BD), dimethyl succinate (DMS), and Ti(OBu)_4_ (TBT) were purchased from Sigma Aldrich. Pripol 1009 was kindly supplied by Croda Italiana S.p.A. (Mortara, Italy).

**Figure 1:**
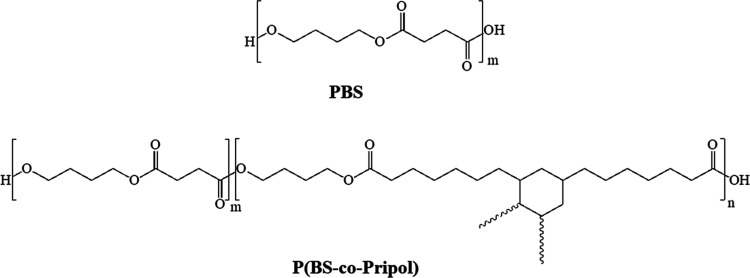
Chemical formula of PBS and P(BS-*co*-Pripol) (the length of Pripol cyclohexane side branches is not fixed, 7/9 carbon atoms each).

The syntheses of the PBS homopolymer and P(BS-*co*-Pripol) copolymer (namely, Co) were conducted via solvent-free two-step polycondensation. In a 200 mL glass reactor, the reagents and the TBT catalyst (200 ppm) were added. The system was placed in a thermostated silicon oil bath and stirred at 100 rpm by a two-bladed centrifugal stirrer equipped with an overhead motor (IKA-Werke GmbH & Co., Staufen, Germany). During the first stage, the temperature was set at 180 and 190 °C (namely, *T*^1st^) for PBS and P(BS-*co*-Pripol), respectively, under a constant nitrogen flow. The conditions were maintained until 90% of the expected amount of methanol was distilled off, about 90 min. During the second stage, the temperature was increased to 230 °C (namely, *T*^2st^), while the pressure was slowly decreased to 0.1 mbar. This last step lasted until a constant value of torque was observed, about 2 h. The products were discharged from the glass reactor and allowed to cool to room temperature. The so-obtained materials appear like beige-colored solids. The molecular formulas and the synthesis details of PBS and P(BS-*co*-Pripol) are reported in Figure 1 and Table 1, respectively.

**Table 1:** Reagent Amounts and Operative Conditions of the PBS Homopolymer

polymer	DMS (mol %)	Pripol 1009 (mol %)	BD (mol %)	*T*^1st^ (°C)	*T*^2nd^ (°C)	*t*^1st^ (min)	*t*^2nd^ (min)
PBS	100		120	180	230	90	120
P(BS-*co*-Pripol)	85	15	120	190	230	90	120

The two repeating units differ for the acid moiety: the succinic subunit being linear and short and the Pripol one being longer with an aliphatic six-carbon ring containing aliphatic branches.

### 2.2 Preparation of TPWF-Based Formulations

**Figure 2:**
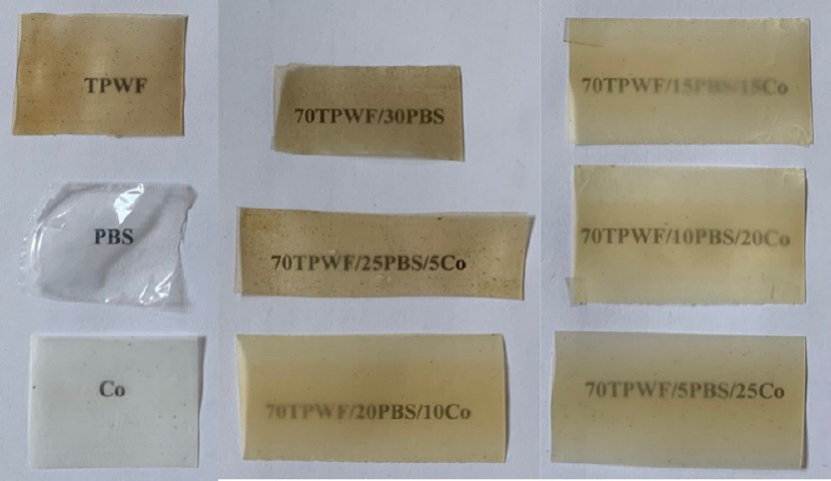
Visual image of TPWF-based films.

Thermoplastic plasticized flour-based films (TPF) were manufactured using a twin-screw microextruder (DSM Explorer 5&15 CC Micro Compounder) provided with a microfilm die and coupled with a line for the cast film (DSM Film Device). Wheat flour and plasticizers were mixed at low speed in a laboratory mixer (planetary mixer, 60 rpm for 3 min). After that, the mixture was introduced in the extruder considering a further mixing at a screw speed of 30 rpm, mixing time of 3 min, and an adequate profile of temperature for the three sections of the extruder (feeding, metering, and die) of 135–140–145 °C. The same temperature profile was adopted for the preparation of TPF blends at different homopolymer/copolymer (PBS/Co) weight amounts. Film strips a few meters long, about 30 mm wide, and 280 μm thick were obtained. Visual images and adopted codes for the produced films are reported in Figure 2 and Table 2, respectively.

**Table 2:** Compositions of TPWF-Based Films

	TPWF	PBS	Co
TPWF	100	0	0
PBS	0	100	0
Co	0	0	100
70TPWF/30PBS	70	30	0
70TPWF/25PBS/5Co	70	25	5
70TPWF/20PBS/10Co	70	20	10
70TPWF/15PBS/15Co	70	15	15
70TPWF/10PBS/20Co	70	10	20
70TPWF/5PBS/25Co	70	5	25

### 2.3 Characterization of the PBS Homopolymer and P(BS-*co*-Pripol) Copolymer

The chemical structures of the synthetized polymers and the composition as well as the repeating unit distribution of the copolymer were determined by ^1^H-NMR and ^13^C-NMR spectroscopy acquired at room temperature with a Varian Inova 400-MHz (Palo Alto, CA, USA). NMR analysis was also employed to determine any composition changes in degraded samples.

Gel permeation chromatography (GPC) was used to measure the molar mass and the polydispersity index. An Agilent 1100 HPLC instrument equipped with a PLgel 5 μm MiniMIX-C column and a refractive index detector was employed (Agilent Technologies, Santa Clara, CA, USA). The system was eluted with chloroform at a rate of 0.3 mL/min, at 30 °C. Polystyrene standards (2000–100,000 g/mol) were used to obtain a molecular-weight calibration curve.

### 2.4 Characterization of TPWF-Based Formulations

The microstructures of the produced formulations were investigated by scanning electron microscope analysis of fractured surfaces (FESEM, Supra 25-Zeiss), obtained by cryofracturing the samples in liquid nitrogen. The surfaces were gold-sputtered in order to provide electric conductivity, and the samples were observed using an accelerating voltage of 2.5 kV.

Thermal characterization of TPWF-based formulations was carried out by both thermogravimetric analysis (TGA) and differential scanning calorimetry (DSC). TGA (Seiko Exstar 6300) experiments were performed for each sample from 30 to 600 °C at 10 °C/min under a nitrogen atmosphere (250 mL/min) in order to evaluate the role of polymer addition in the thermal stability of the TPWF matrix.

DSC (DSC-TA instrument, Q200) measurements (first heating, cooling, and second heating scans) were performed in the temperature range of −50 to 150 °C, at a heating/cooling rate of 10 °C/min. Three specimens were used to characterize each material.

Tensile tests of neat TPWF and TPWF-based systems were performed to evaluate the effect of PBS addition on the mechanical response of the TPWF matrix. The tests were performed using a universal test machine (Lloyd Instruments, LR30KPlus) according to the UNI ISO 527 on 20 × 150 mm rectangular specimens about 280 μm thick. Young’s modulus (*E*), maximum stress (σ_max_), strain at maximum stress (*e*_max σ_), stress at break (σ_b_), and elongation at break (ε_b_) were calculated from the resulting stress–strain curves with software specific to the test machine: NEXYGENPlus Materials Testing. The measurements were done at room temperature, and at least five specimens for each formulation were tested.

Surface wettability of the TPWF-based films was studied through static advancing water contact angle measurements with a standard goniometer (FTA2000, First Ten Angstroms, Inc., Portsmouth, UK) equipped with a camera, and drop shape analysis (SW21; FTA32 2.0 software, First Ten Angstroms, Inc., Portsmouth, UK) was used to test the water contact angle at room temperature. The contact angle was determined by randomly putting five drops of distilled water (2 μL) with a syringe onto the film surfaces, and after 1 s, the average values of five measurements for each drop were used.

Disintegrability in composting conditions was carried out following the European standard ISO 20200:2015. The test determines, on a laboratory scale, the degree of disintegration of plastic materials under simulated intensive aerobic composting conditions. The degree of disintegration *D* was calculated in percent by normalizing the specimen weight at different days of incubation to the initial weight using eq 1
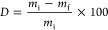
1where *m*_i_ is initial dry plastic mass, *m*_f_ is dry plastic material mass after the test.

Films of dimension 15 mm × 15 mm (mean thickness, 280 μm) were weighed and buried into the organic substrate at 4–6 cm depth in the perforated boxes guaranteeing the aerobic conditions and incubated at 58 °C and 50% humidity. The systems can be considered disintegrable according to the European standard when 90% of the plastic sample weight is lost within 90 days of analysis. In order to simulate the disintegrability in compost, a solid synthetic waste was prepared, mixing sawdust, rabbit food, compost inoculum supplied by Genesu S.p.a., starch, sugar, oil, and urea. The specimens tested were taken out at different times (3, 7, 10, 14, 21, 28, 42, 56, 77, and 90 days), washed with distilled water, and dried in an oven at 37 °C for 24 h. The surface microstructures of Co and TPWF-based systems before the composting and at different days (21, 56, and 90 days) of incubation were investigated by field-emission scanning electron microscopy (FESEM), while the photographs of the specimens were taken for visual comparison.

## 3 Result and Discussion

### 3.1 Homopolymer and Copolymer Characterization

**Figure 3:**
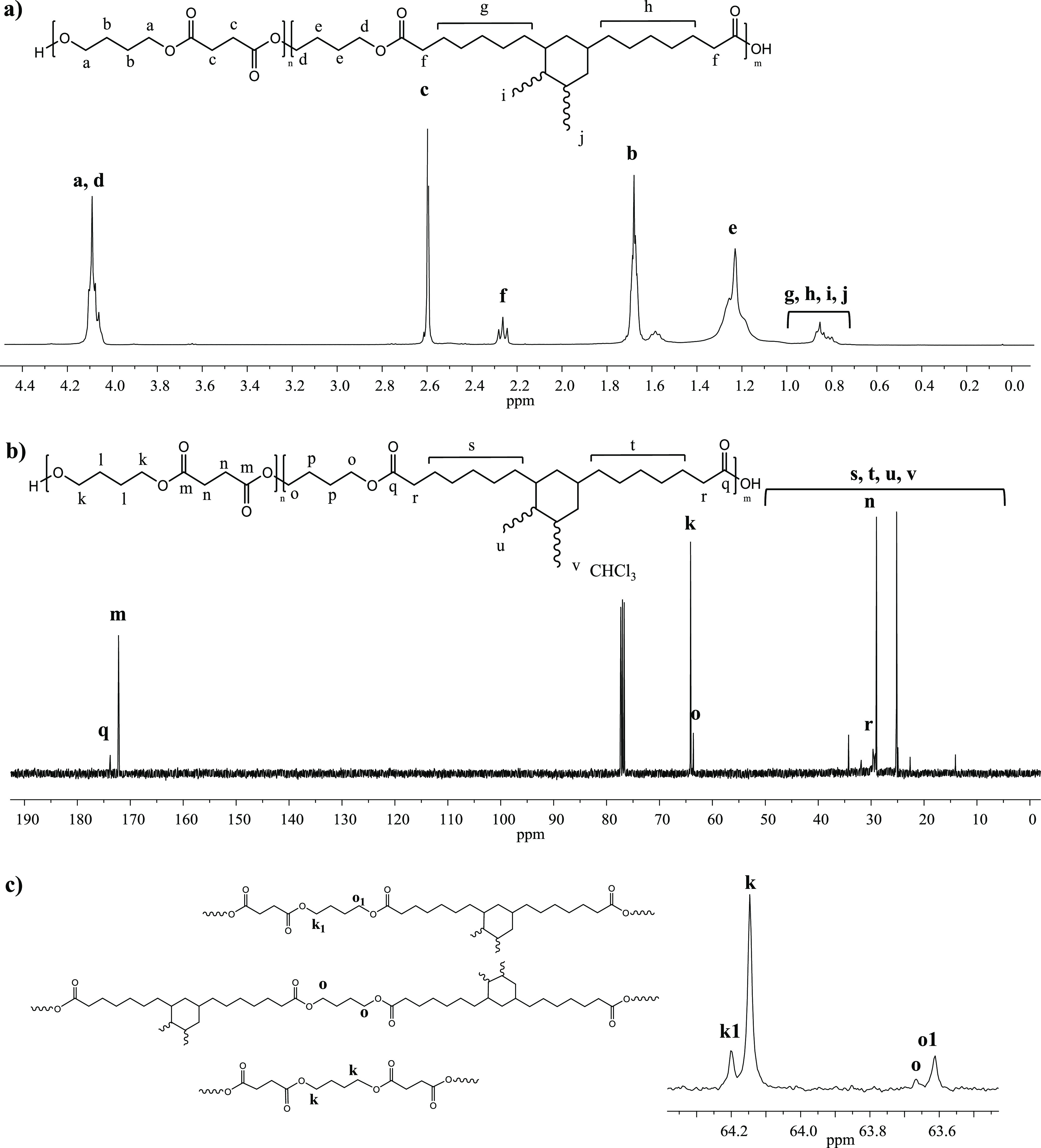
^1^H-NMR (a) and ^13^C-NMR (b) spectra of P(BS85BPripol15) with the peak assignment. Magnification of the ^13^C-NMR spectrum in the 64.4–63.5 ppm region (c) and schematic representation of S–B–Pripol, Pripol–B–Pripol, and S–B–S triads.

The chemical structures and copolymer compositions of the synthesized samples have been determined by ^1^H-NMR spectroscopy, while the chemical architecture of the copolymer has been evaluated by ^13^C-NMR analysis. The relative data are collected in Table 3. The chemical shift peaks for the PBS homopolymer (data not shown) are as follows: δ 4.2 ppm (t, 4H); δ 2.6 ppm (s, 4H); δ 1.7 ppm (t, 4H). In Figure 3 are the reported ^1^H-NMR and ^13^C-NMR spectra of the P(BS-*co*-Pripol) sample together with the peak assignment. For both polymers, no additional peaks have been detected allowing the exclusion of the occurrence of side reactions during the synthesis process.

**Table 3:** Chemical Characterization Data of the PBS Homopolymer and P(BS-*co*-Pripol) Copolymer

polymer	S (mol %)	Pripol (mol %)	*M*_n_ (g/mol)	PDI	*b*	*L*_BS_	*L*_BPripol_
PBS	100		52,000	2.0			
P(BS-co-Pripol)	82	18	46,000	2.4	0.98	5.0	1.3

The actual composition of the copolymer has been calculated from the normalized area of the c and f peaks in the ^1^H-NMR spectrum (Figure 3a) corresponding to succinic and Pripol moieties, respectively (Table 2). The degree of randomness (*b*) and the block length (*L*) have been calculated considering the ^13^C-NMR^28,31,32^ (Figure 3b), in particular the region in between 64.4 and 63.5 ppm (Figure 3c) where the carbon atoms of the −OCH_2_– group are located. In this range of ppm, four different signals are observed: the k peak corresponding to S–B–S triads, the o peak due to Pripol–B–Pripol triads, and the k_1_ and o_1_ peaks related to the S–B–Pripol triads. The degree of randomness was determined through the equation
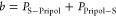
2with *P*_S–Pripol_ being the probability of finding an S subunit next to a Pripol one and *P*_Pripol–S_ being the probability of finding a Pripol subunit next to an S one. *P*_S–Pripol_ and *P*_Pripol–S_ can be expressed as follows
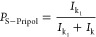
3
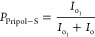
4where *I*_k_, *I*_k_1__, *I*_o_1__, and *I*_o_ represent the integrated intensities of the resonance peaks of the S–B–S, S–B–Pripol, Pripol–B–S, and Pripol–B–Pripol triads, respectively.

Additionally, the average lengths of the BS and BPripol blocks in the copolymer chain are defined as
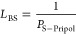
5
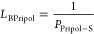
6

The degree of randomness appeared to be very close to 1, indicating a random distribution along the macromolecular chain of BS and BPripol repeating units, and the average block lengths of the BS and BPripol sequences (*L*_BS_ and *L*_BPripol_), reported in Table 2, are directly proportional to the molar amounts of succinic and the Pripol subunits contained in the polymer backbone. In Table 2, molecular weight data (*M*_n_) are also collected. GPC analysis revealed high and comparable molecular weight for both the synthetized polymers and polydispersity indexes compatible with the synthetic strategy adopted. It is worth highlighting that high *M*_n_ is necessary for the material processing.

### 3.2 Morphological Properties of TPWF-Based Formulation

**Figure 4:**
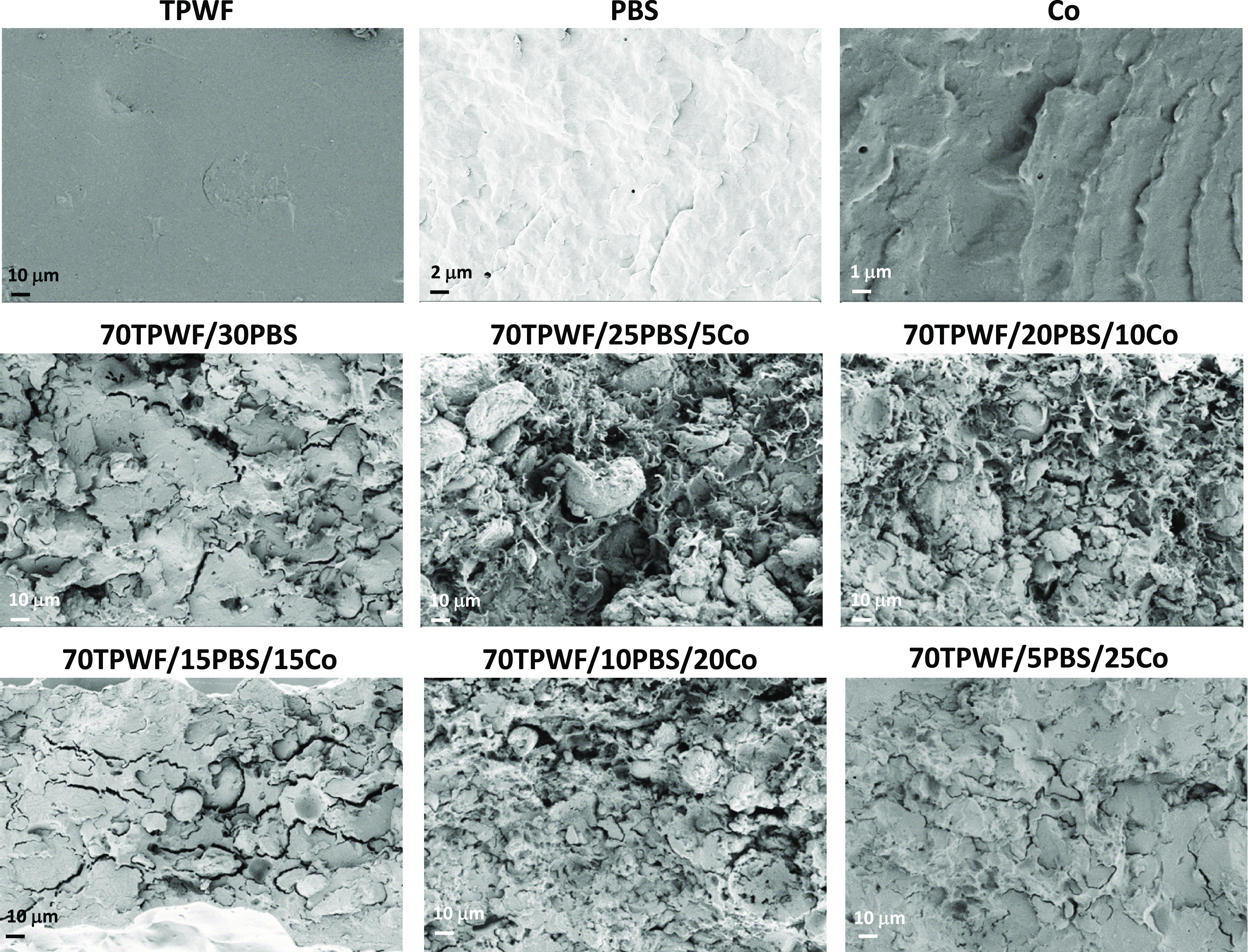
FESEM images of fractured cross sections of TPWF, PBS, Co, and TPWF-based films.

The morphologies of fractured surfaces for TPWF/polymer blends were observed by FESEM (Figure 4), and differences were found as a function of blend composition: in detail, TPWF exhibited a continuous and homogeneous surface, while PBS and Co showed rough surfaces, with a semiductile fracture behavior. The morphological variations for blends may be associated with the differences in the viscosity of polymers and TPWF along with the varying hybrid compositions. No visible starch granules can be detected on the fracture surface of TPWF/polymer blends, even if the incompatible sea-island bi-phase structure was noted in the case of 70TPWF/30PBS. Clear phase separation was indeed observed on the fractured surface, with TPWF as the continuous phase and PBS as the dispersed phase, with formation of microcavities at the filler–matrix interface, suggesting poor compatibility. In the ternary systems, the disappearance of matrix fibrillation from the fracture surface from low content of Co up to the highest value is notable due to the compatibilizer effect of Co. Besides that, different surface roughness profiles were achieved, essentially due to diverse interaction of the polymeric phase with starchy particles of the plasticized flour. In detail, dimensional reduction of the conglomerates and better dispersion of the starchy particles were obtained with increasing content of Co, in perfect line with the results of mechanical and wettability characterization presented below. Additionally, there is no clear delamination from the polymer matrix, confirming the improved filler–matrix interactions in the Co compatibilized blends.

### 3.3 Thermal Analysis of TPWF-Based Formulations

#### 3.3.1 TGA Analysis

**Figure 5:**
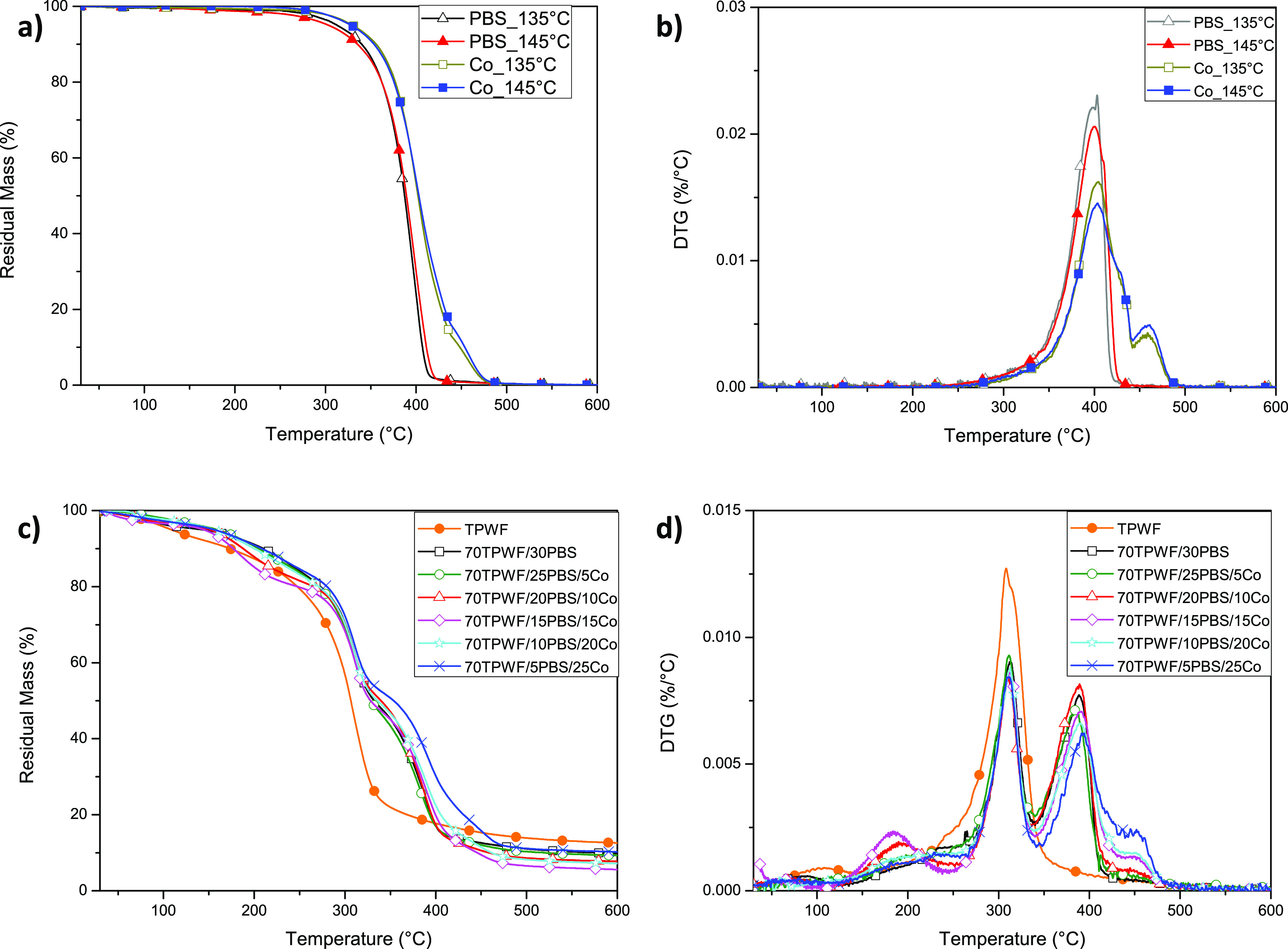
Residual mass (a,c) and derivative mass loss (b,d) curves of PBS and Co processed at 135 and 145 °C (TG (a) and DTG (b)) and TPWF-based films (TG (c) and DTG (d)).

Thermogravimetric analysis of the blends was carried out. Figure 5a,b shows the curves and the derivative mass loss curves as a function of temperature for neat PBS and random copolymer Co, processed at 135 and 145 °C, while Figure 5c,d shows the profiles for the different TPWF/polymer blends.

The PBS homopolymer undergoes thermal degradation in a single step, as visible in Figure 5b, with no significant mass loss until about 300 °C and complete decomposition around 430 °C, which is in agreement with the literature data: the primary mass loss is caused by the volatilization of small molecules, including succinic acid and butylene glycol, followed by major thermal degradation of PBS chains, due to random chain scission at the ester bonds, with the formation of carboxylic and vinyl end groups.^33,34^ In the case of P(BS-*co*-Pripol), Co, the weight loss curve is shifted at higher temperature and the degradation takes place in two steps: the first one is proportional to the amount of BS co-units, and the second one is related to the content of BPripol sequences. The thermal stability improvement is consequent to the introduction of the Pripol subunit along the PBS macromolecular chain, determining a reduction of the easily thermally degradable ester groups that, in turn, are replaced with quite long and thermally more stable PE-like segments. Concerning the effect of the processing temperature, no appreciable differences can be evidenced on the thermal stability by changing this parameter during the sample preparation, from 135 to 145 °C. This evidence confirms that no thermal degradation occurs under the processing conditions (temperature and time).

In the presence of the thermoplastic wheat flour, three distinct regions can be evidenced in the TGA curves (Figure 5c). The initial weight loss is generally due to the loss of volatiles (water), reduced in the case of blends, indicating a limited moisture absorption in comparison with neat TPWF. After that, a visible further decrease in the 125–250 °C range was noted, specifically for the blend systems containing 15 and 20 wt % PBS homopolymer, that could be ascribed to volatilization of excess glycerol. After the evaporation of plasticizers, the TPWF fraction started to degrade, with an onset degradation temperature that moved from 232 °C for neat TPWF up to 268 °C for the different ternary systems. Even the peak of derivative weight loss, related to the superposition of PBS and flour fraction, was slightly shifted from 306 °C for the material having no polymer fractions to 313 °C in the case of systems containing Co and PBS at different weight contents. The final stage represents the main degradation zone of PBS and Co (above 400 °C), which were in line with the DTG degradation profiles observed in Figure 5b. The TGA results, in particular the variation of onset degradation temperature and maximum degradation temperature, showed a thermal stability improvement by blending TPWF with PBS or Co, in this last case to a greater extent.

#### 3.3.2 DSC Analysis

**Figure 6:**
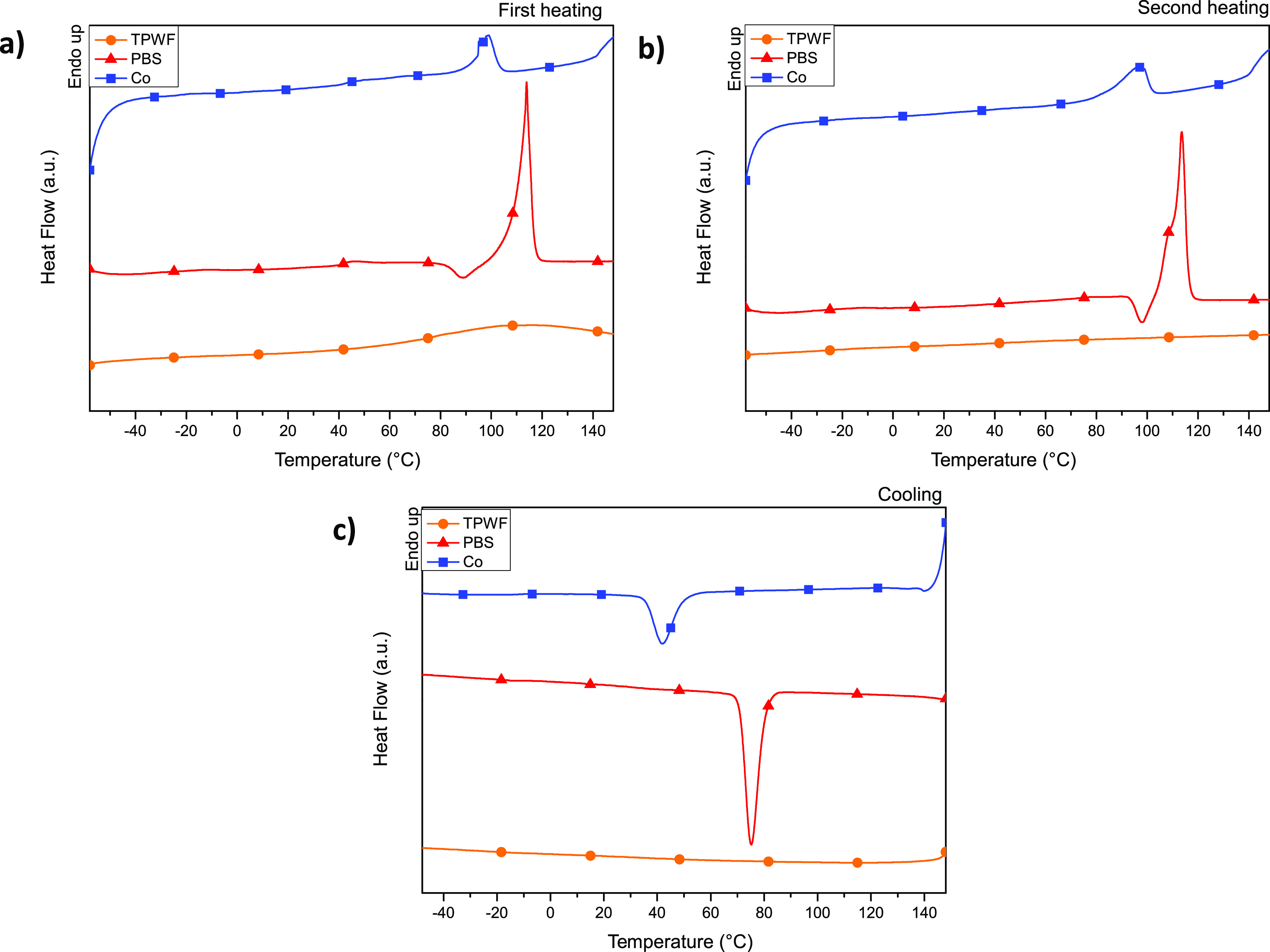
DSC thermograms (first heating scan (a), second heating scan (b), and cooling scan (c)) of TPWF, PBS, and Co films.

Figure 6 shows the calorimetric traces of TPWF and the two polymer matrices, PBS and Co, subjected to extrusion process. The TPWF first scan calorimetric trace shows a wide endothermic peak due to water release by evaporation. No signals in the following cooling and heating scans have been detected for TPWF, indicating that no thermal transitions take place. The first DSC scan profiles of both PBS and Co (Figure 6a) are typical of semicrystalline polymers, being both characterized by both the glass-to-rubber transition step at low temperature and the endothermic melting peaks at higher temperature. In the case of the PBS homopolymer, just before the melting phenomenon, at 90 °C, a little intense exotherm coming from the crystallization of smaller crystals can be detected. In particular, the glass transition temperature of PBS located at −35 °C is reduced by copolymerization, reaching a value of −42 °C in the random copolymer. The longer acid co-unit, in fact, increases the macromolecular mobility thanks to the PE-like segments of the Pripol moiety and to the branches on the aliphatic ring, exerting a plasticizing effect. The PBS melting phenomenon, as a consequence of the introduction of Pripol co-units, undergoes changes, being the homopolymer melting process characterized by a single peak at 114 °C, which in the copolymer moves at lower temperature (97 °C). Together with the *T*_m_ decrease, copolymerization also determines the reduction of the melting enthalpy Δ*H*_m_, i.e., a less intense endotherm, indicating a lower amount of crystal phase in Co that, in addition, is characterized by a lower degree of perfection. Moreover, copolymerization leads to the disappearance of the exotherm centered at 90 °C in the PBS trace. After melting, the polymers have been cooled down at 10 °C/min, and the corresponding DSC curves are reported in Figure 6b. As one can see, both samples crystallize during the cooling step as indicated by the exotherms at 75 and 44 °C for PBS and Co, respectively. As one might expect, the co-unit introduction slows down the crystallization process. The second DSC heating scan for the PBS homopolymer (Figure 6c) is practically the same as the first, while for Co, an enlargement and a slight shift of the endothermic peak position toward lower temperature are detected, indicating a further decrease of crystalline perfection portion during the cooling step.

**Figure 7:**
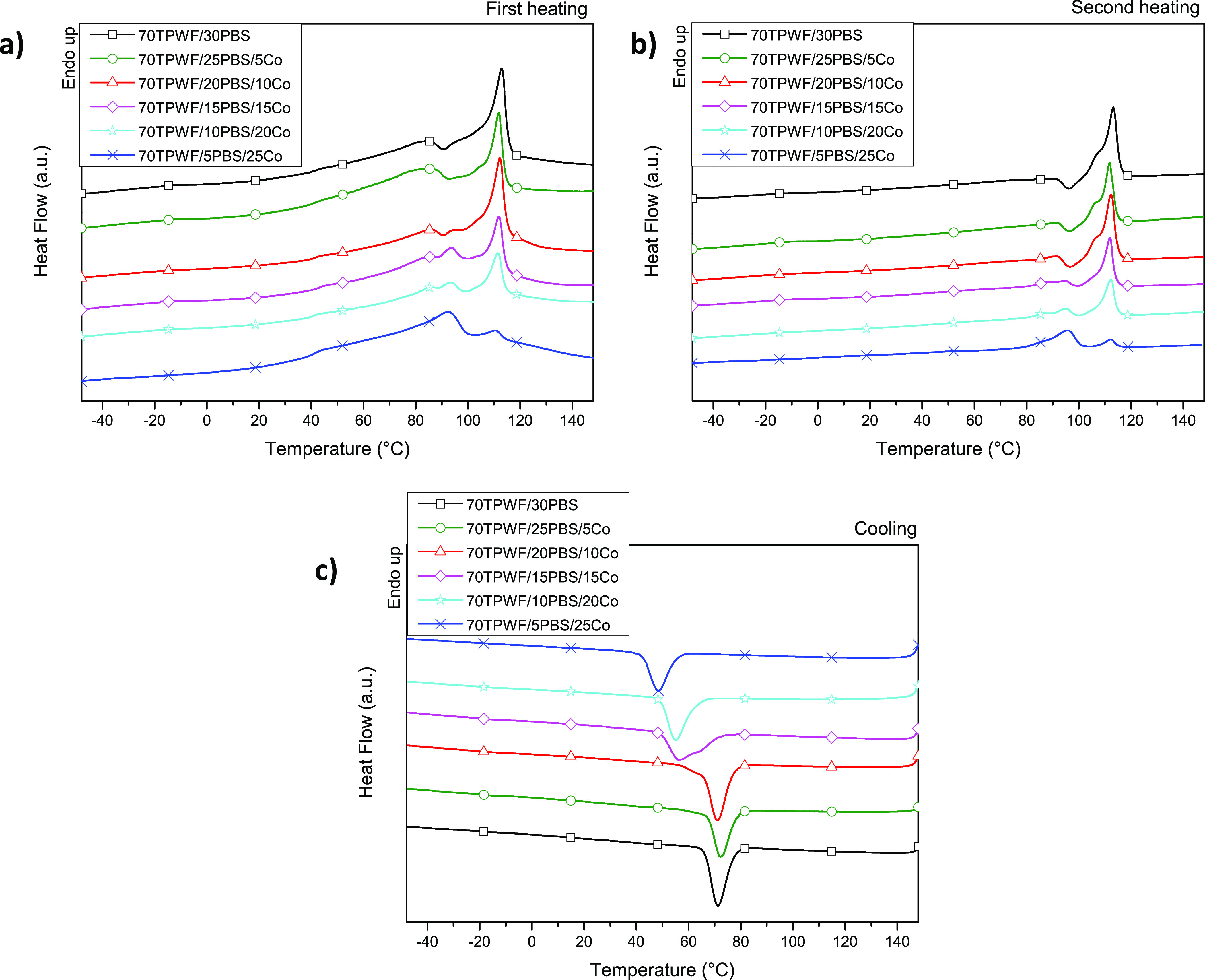
DSC thermograms (first heating scan (a), second heating scan (b), and cooling scan (c)) of TPWF-based films.

In Figure 7, the DSC curves of the TPWF/polymer blends are reported. As one can see, the presence of the signals of all the components in the blend can be detected, with the corresponding intensity being proportional to their amount. The first heating scan traces are very similar to the second scan ones; nevertheless, the as-extruded ternary blends present the wide endothermic peak due to water release of the TPWF, underlying the melting peaks of the polymer matrix. As a consequence, the calculation of the temperature (*T*_c_, *T*_m_) and enthalpy (Δ*H*_c_, Δ*H*_m_) values of the polymer thermal phenomena, occurring during cooling and heating scans, has been carried out after water removal, i.e., in the cooling and second heating scans, and results are reported in Table 4.

**Table 4:** DSC Characterization Data of Ternary Blends: Cooling Step from the Melt and Subsequent Heating Scan

	cooling		2nd heating				
sample	*T*_c_ (°C)	Δ*H*_c_ (J/g)	*T*_g_ (°C)	*T*_cc_ (°C)	Δ*H*_cc_ (J/g)	*T*_m_ (°C)	Δ*H*_m_ (J/g)
70TPWF/30PBS	71	21	–48	97	4	113	24
70TPWF/25PBS5Co	71	16	–49	97	3	112	22
70TPWF/20PBS10Co	70	22	–49	96	3	112	20
70TPWF/15PBS15Co	56	18	–49			94	2
						112	14
70TPWF/10PBS20Co	55	16	–50			94	4
						112	11
70TPWF/5PBSCo	48	15	–50			94	14
						112	4

The crystallization capability of the polymer matrix during the cooling step (Figure 7b) is not compromised by the TPWF presence, being all the blends characterized by comparable crystallization enthalpy values (Table 4). Nevertheless, a progressive displacement of the exothermic peaks toward lower temperature from 71 °C for 70TPWF/30PBS to 48 °C for 70TPWF/5PBS/25Co is observed. Moreover, in the blends containing comparable amounts of PBS and Co, two overlapping exotherms can be evidenced, originating from BS sequence crystallization in the homopolymer and the copolymer, respectively. Concerning the second heating scan (Figure 7c), as mentioned above, it results very similarly in the first one, with the only difference that the peak of water evaporation is no longer present. The presence of TPWF determines a decrease of the glass-to-rubber transition temperature evidencing its plasticizer effect. As temperature increases, the PBS-rich blends exhibit the crystallization peak at around 97 °C (as observed for the neat homopolymer) followed by the melting endotherm centered at 112 °C, with a shoulder at lower temperature (105 °C) ascribable to the melting of the less perfect crystal fraction. As the Co amount rises, the exotherm at 97 °C is replaced by an endothermic peak (94 °C), ascribable to the melting of the Co crystalline phase, whose intensity grows at the expense of the PBS crystals melting at higher temperature.

### 3.4 Tensile Behavior of TPWF-Based Formulations

**Figure 8:**
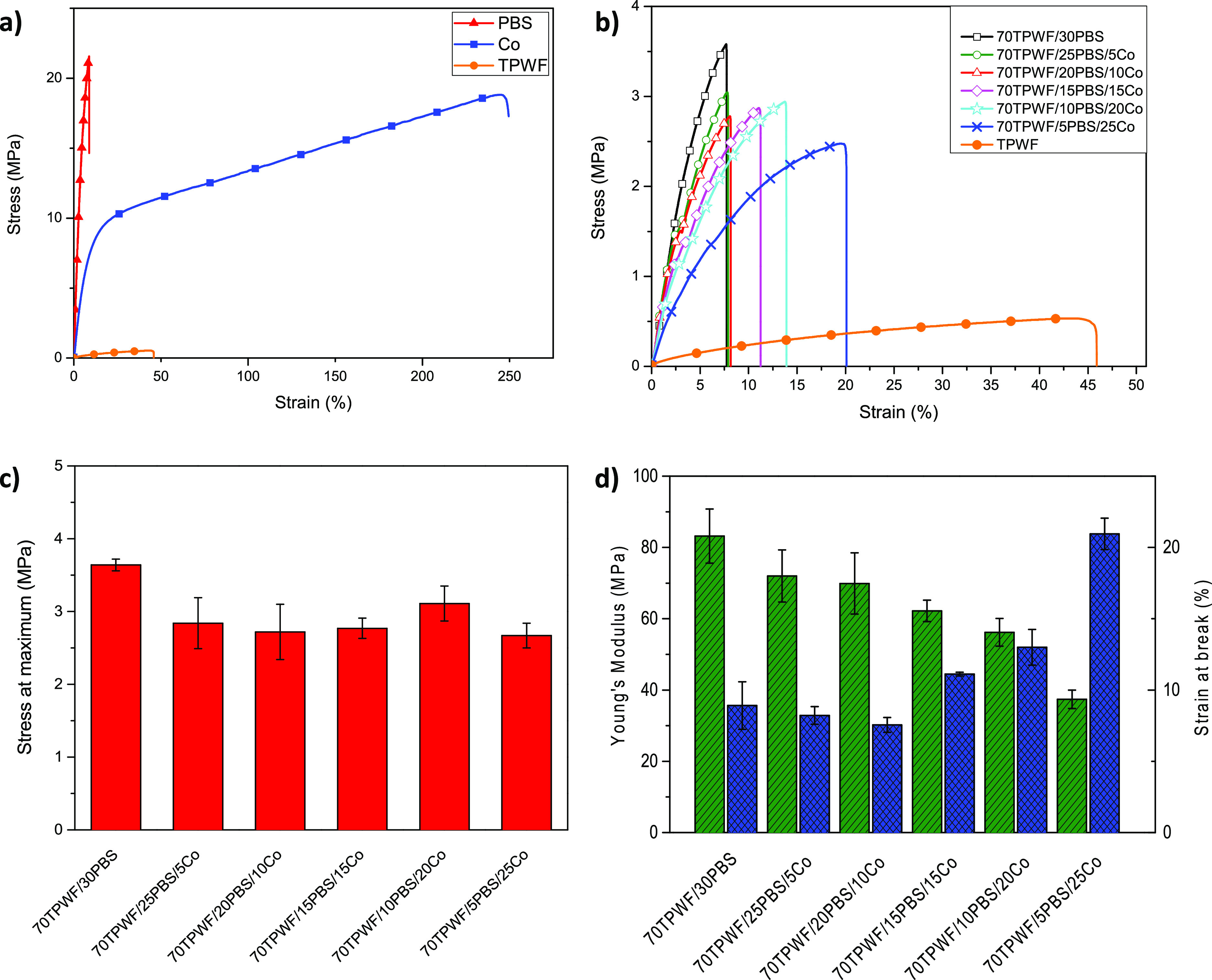
Stress–strain curves of TPWF, PBS, and Co films (a) and TPWF-based films (b) and stress at maximum (c) and stress at break and Young’s modulus (d) of TPWF-based films.

Table 5 shows the characteristic values of the tensile tests. In Figure 8, the graphs of the tensile tests carried out on the three polymeric matrices (PBS, Co, and TPWF) highlight the characteristic mechanical behavior of each material; the PBS homopolymer shows high elastic modulus and tensile strength values but modest deformation, which ends with a brittle fracture. The TPWF shows much lower mechanical performances, both in terms of the modulus and tensile strength, while it deforms plastically 5 times more than the PBS. The obvious differences in mechanical behavior give evidence of the low compatibility between PBS and TPWF, already observed by FESEM investigation. Co shows an intermediate behavior between the two previous ones, showing good Young’s modulus and stress resistance values with tough behavior and high deformation capability.

**Table 5:** Parameters from Tensile Tests for the TPWF-Based Films

	Young’s modulus, *E* (MPa)	maximum stress, σ_max_ (MPa)	strain at maximum stress, ε_max σ_ (%)	stress at break, σ_b_ (MPa)	strain at break, ε_b_ (%)
TPWF	4.7 ± 0.1	0.6 ± 0.1	45.8 ± 4.9	0.32 ± 0.1	48.4 ± 4.7
PBS	402 ± 23	22.7 ± 1.6	10.8 ± 2.8	22.6 ± 1.6	10.8 ± 2.8
Co	120 ± 5	18.4 ± 1.4	240 ± 36	12.2 ± 3.5	253 ± 34
70TPWF/30PBS	83.2 ± 7.6	3.6 ± 0.1	8.8 ± 1.5	3.5 ± 01	8.9 ± 1.7
70TPWF/25PBS/5Co	72.0 ± 7.3	2.9 ± 0.4	8.1 ± 0.6	2.8 ± 0.4	8.2 ± 0.6
70TPWF/20PBS/10Co	69.9 ± 8.6	2.7 ± 0.4	7.5 ± 0.5	2.6 ± 0.3	7.6 ± 0.5
70TPWF/15PBS/15Co	62.2 ± 3.0	2.8 ± 0.1	11.0 ± 0.1	2.6 ± 0.2	11.1 ± 0.1
70TPWF/10PBS/20Co	56.2 ± 3.9	3.1 ± 0.2	12.9 ± 1.2	3.0 ± 0.3	13.0 ± 1.3
70TPWF/5PBS/25Co	37.4 ± 2.6	2.7 ± 0.2	20.5 ± 1.1	2.52 ± 0.2	21.0 ± 1.1

The formulations with a prevalence of TPWF are intended to achieve low-cost mixtures, the addition of PBS to improve performance while maintaining the eco-sustainability characteristics of the final materials produced, and the further introduction of Co to enhance the TPWF/PBS chemical and physical compatibility. The mixtures of TPWF with different amounts of the two synthetic polymers have the purpose of correlating the PBS/Co ratio with the mechanical behavior of the blends. Table 5 reports the results of tensile tests performed on neat samples and TPWF-based formulations. As one can see from Figure 8b–d, the addition of the 30 wt % PBS homopolymer (70TPWF/30PBS) produces a significant increase in resistance compared to neat TPWF; however, the deformation values remain low as for the neat PBS. In the 70TPWF/25PBS/5Co and 70TPWF/20PBS/10Co blends, the substitution of 5 and 10 wt % PBS homopolymers with the copolymer produces exclusively a modulus and resistance reduction without improvement of deformability. The mixtures with 15 and 20 wt % Co (70TPWF/15PBS/15Co and 70TPWF/10PBS/20Co) show a progressive increase of deformation preserving good values of elastic modulus and stress. The formulation with TPWF and 25 wt % Co (70TPWF/5PBS/25Co) shows the greatest deformation of the mix set (≈21%) but also a slight reduction in strength. The results obtained suggest that PBS confers strength and rigidity to the mixture, while Co acts both as the reinforcement and toughener. The Co material also acts as the link between the rigid and fragile behaviors of PBS and the low resistance and plastic deformation response to stress of TPWF. In line with the results of a previous paper from us,^29^ where we have already observed that a system based on thermoplastic wheat flour mixed with polycaprolactone in a ratio of 75:25, without any compatibilizing agent, showed a high level of deformation (450%) (although not connected with a satisfactory mechanical behavior (tensile strength, 5 MPa)), we can here conclude that the approach of substituting a great part (more than 70 wt %) of the polymeric phase in a blend can certainly represent an acceptable compromise and a satisfactory solution to the problem of time limited use of fossil-based sources in the packaging sector. Nevertheless, the comparison with the reference TPWF (tensile strength (1.1 ± 0.1 MPa) and deformation at break (75 ± 6, %)) clearly indicated that the adopted solution could represent a challenge to the improvement of limited performance of flour-based biopolymers. Chabrat et al.^35,36^ also successfully produced wheat flour/PLA blends. In this case, it has been proven that adding 20 parts of PLA to plasticized wheat flour (83.3% wheat flour (at its equilibrium humidity) and 16.7% glycerol) increased its Young’s modulus up to 709 MPa and decreased its elongation at break to 7%. The use of citric acid was also investigated: it was verified that compatibility of the phases, depending on the use of extra water (or not) during the extrusion, was promoted, in a competition between glycerol and citric acid for the plasticization.

Anyway, these are the sole cases present in the literature where a prevalent fraction of plasticized flour was mixed with a biopolymeric matrix and adequate tensile strength and ductility were definitely achieved.

Looking at the comparison with PBS based blends, as in the case of substitution of thermoplastic starch with thermoplastic flour, we should underline that good mechanical performance was, in general, reached to the expense of either high refining costs for the starch-based component or higher content of PBS fraction, which always surpassed the weight content of the plasticized flour. In the paper of Yin et al.^19^ it was demonstrated that tensile strength and elongation at break of TPS/PBS (60/40) blend were only 6.4 MPa and 4%, respectively, and the best results were obtained only when the inversion of the TPS:PBS ratio (40/60) was considered. The direct comparison with the results from our investigation highlighted that, even at a prevailing amount of plasticized not refined starches, acceptable properties for films to be used in the packaging sector can be reached.

Nevertheless, none of the cited works investigated in depth the compost disintegration of the produced films, which is extremely important when the materials are intended for packaging use. According to this, we extended the research to the evaluation of produced materials’ attitude to undergo compost disintegration.

### 3.5 Simulated Disintegration in Compost

**Figure 9:**
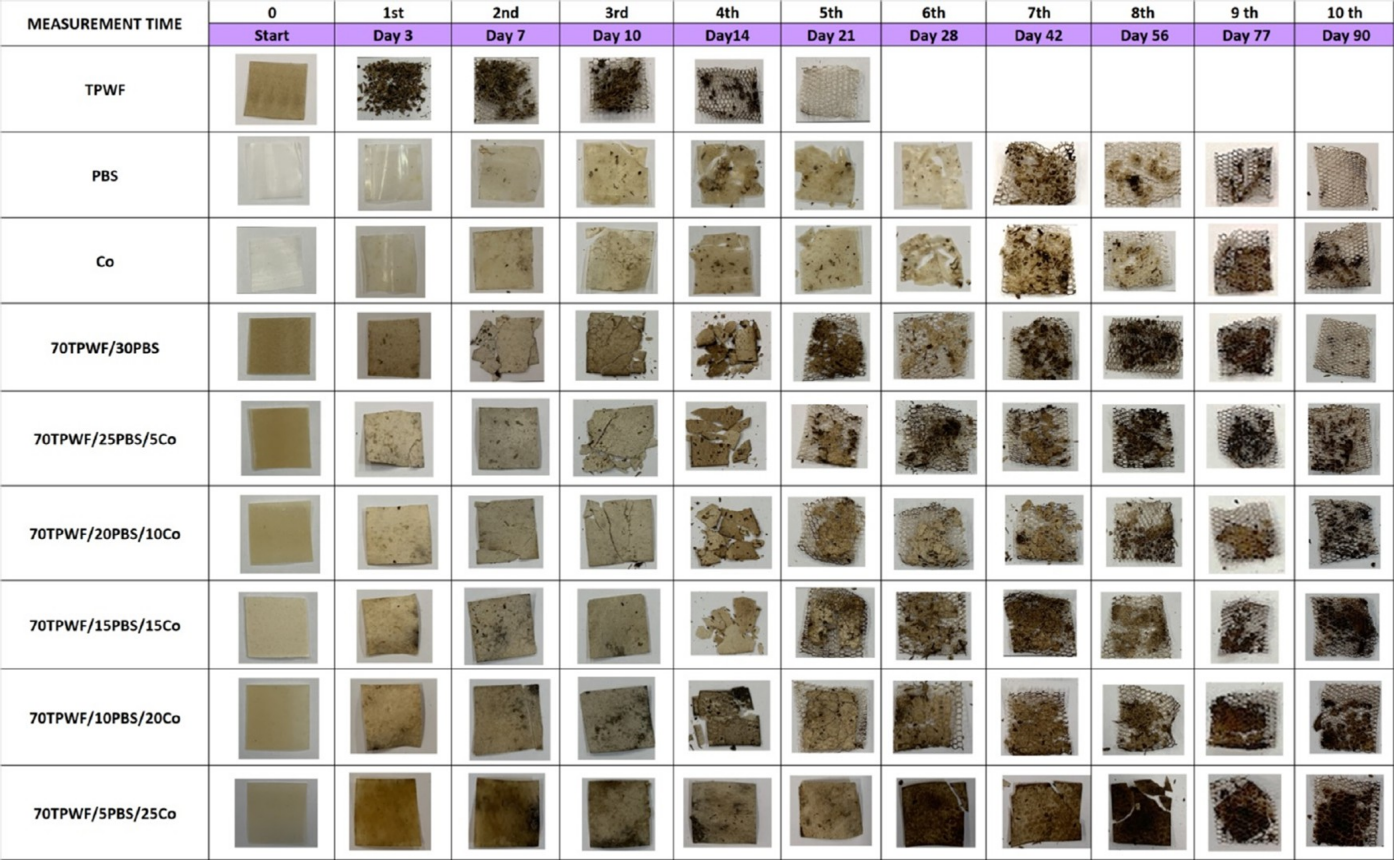
Visual observation of TPWF, PBS, Co, and TPWF-based films before and after different days in composting conditions.

**Figure 10:**
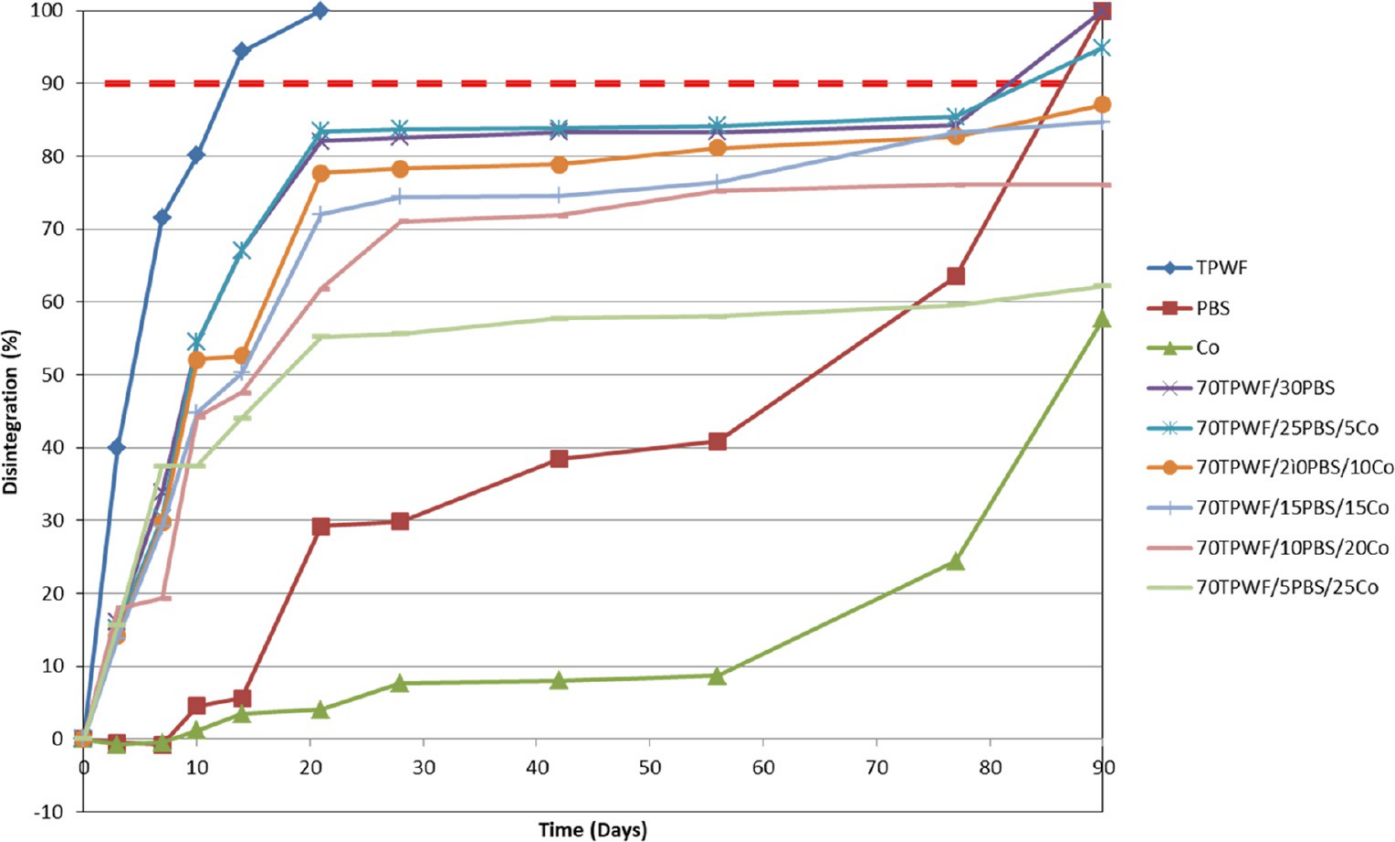
Disintegrability values of TPWF, PBS, Co, and TPWF-based films before and after different days in composting conditions.

The post-use performance of TPWF- and PBS-based formulations was evaluated by performing disintegrability tests under composting conditions, according to the EN ISO 20200:2015. Figure 9 shows the images of all different TPWF-based films at the initial time (before the disintegration in composting conditions) and after several incubation times, while Figure 10 exhibits the disintegration values at different incubation times on a laboratory scale. It was observed that all samples changed their color and dimensions just after the first days of incubation, especially for neat TPWF film and TPWF-based formulations, consequent to the hydrolytic degradation. Neat TPWF films after only 3 days appeared completely fractured, with the behavior being related to the hydrophilic nature of flour-based films that facilitates the water attack.

The disintegration kinetics of PBS and Co films are completely different with respect to neat TPWF being, in general, lower. Nevertheless, the polymer composting is accelerated in the binary and ternary blends. This is due to the presence of a very high amount of hydrophilic groups in the TPWF fraction of the composites, which easily absorb water and microorganisms from the soil and increase the contact area of the water and microorganisms with the polymer matrix. This phenomenon makes it easier for PBS and Co to hydrolyze and disintegrate and, consequently, increases the weight loss.^37^ The TPWF film completely disintegrated after 14 days of incubation, while after the same time of incubation, PBS and 70TPWF/5PBS/25Co (the formulation with the highest Co content) films reached 6 and 4% disintegration, respectively. The disintegration of the PBS phase is essentially due to the hydrolysis of the amorphous regions, where the attack of microorganisms and fungi present in the soil on the film surface is facilitated.^37,38^ The reduced disintegration value for Co, despite its higher amorphous fraction (i.e., lower crystallinity degree), can be explained on the basis of the lower amount in the Co backbone of hydrolysable −COOR– groups per chain unit, as a consequence of the replacement in the copolymer of 18 mol % succinic moieties with the long Pripol one. The preferential attack by the microorganisms of the −COOR– groups mainly present in the BS segments determines an increase of the BPripol sequence molar fraction, up to 20% with respect to the initial value, in the partially degraded ternary blends. The enrichment of degraded samples in BPripol sequences is in line with a previous study.^28^ Consequently, in TPWF/PBS-based formulations, the disintegrability values decrease with increasing amount of Co. After 90 days, TPWF, PBS, 70TPWF/30PBS, and 70TPWF/25PBS/5Co disintegrated (reaching disintegrability values close to 90%). At the end of the test, at 90 days of incubation in composting conditions, the Co neat film reached 58% disintegrability, while the ternary blends in which the Co amount is progressively increased, 70TPWF/20PBS/10Co, 70TPWF/15PBS/15Co, 70TPWF/10PBS/20Co, and 70TPWF/5PBS/25Co, reached 87, 85, 76, and 62% disintegration, respectively.

### 3.6 Morphological Analysis of Disintegrated Samples

**Figure 11:**
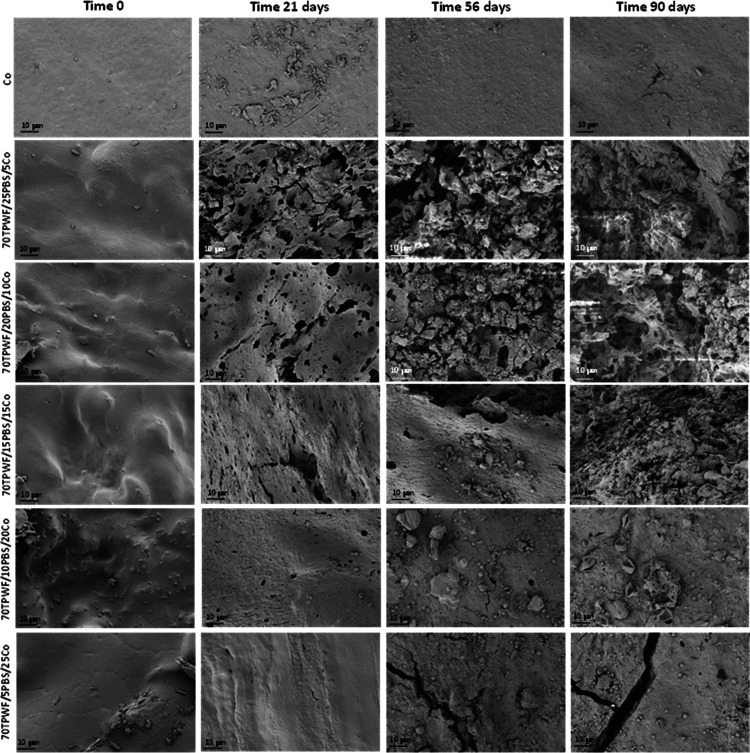
FESEM images of Co and TPWF-based film surfaces before and after different times (21, 56, and 90 days) in composting conditions.

The surface morphology of the partially degraded films was determined via FESEM observations (see Figure 11). At time 0, the neat Co film showed a homogeneous and smooth surface, without the presence of pores or cracks, as already described in the case of plasticized flours.^39^ As reported in the literature, poor miscibility is reported between PBS and thermoplastic starch,^40^ so in our case, the increasing amount of Co (up to 15 wt %) was indeed beneficial for the formation of a matrix-dispersed phase type in TPWF/PBS blends. Nevertheless, at higher amounts, the Co addition produced the appearance of small surface asperities that probably came from not-perfectly plasticized granules.^26,41^

After 21 and 56 days of disintegration in composting conditions, in the case of 70TPWF/25PBS/5Co and 70TPWF/20PBS/10Co samples, the surface showed large smooth areas containing holes with quite sharp boundaries, coming from the fast degrading TPWF majority phase in the presence of slow degrading PBS. The holes arose and became increasingly deeper with increasing erosion time, up to the end of the test at 90 days, where evident porosity connected the holes.^42^ The delay of disintegration time in the presence of higher amounts of Co (from 15 to 25 wt %) was also evident by the loss in porosity detected for 70TPWF/15PBS/15Co, 70TPWF/10PBS/20Co, and 70TPWF/5PBS/25Co, which substantially maintained a relatively smooth surface (only a few holes and cracks appeared), in line with the disintegration profile of neat Co.^43^ These results were in good agreement with weight loss experiments, which proved the expected slower disintegration of TPWF/PBS blends after the addition of Co.

### 3.7 Water Contact Angles of TPWF-Based Formulations

Results of water contact angle (WCA) measurement for TPWF/PBS samples are reported in Table 6. We measured a mean value of 55° for neat TPWF, confirming its inherent hydrophilic behavior, as already observed by Averous et al.^44^ and Mahieu et al.,^45^ who found values ranging from 32 to 53 °C for wheat starch films formulated with varying amounts of water/glycerol ratio. From WCA analysis, it can be shown the high hydrophobic character of the PBS homopolymer in comparison with the TPWF, as expected on the basis of their chemical structure. In the case of Co, the introduction of the large nonpolar Pripol moiety in the repeating unit further increases the material hydrophobicity, increasing the angle value from 59 (for neat PBS) to 63°.

**Table 6:** Water Contact Angles of the TPWF-Based Films

	WCA (°)
TPWF	54.7 ± 1.4
PBS	58.9 ± 1.9
Co	63.5 ± 0.8
70TPWF/30PBS	60.6 ± 1.2
70TPWF/25PBS/5Co	79.2 ± 1.6
70TPWF/20PBS/10Co	79.9 ± 1.9
70TPWF/15PBS/15Co	72.3 ± 0.8
70TPWF/10PBS/20Co	76.9 ± 1.7
70TPWF/5PBS/25Co	70.4 ± 1.9

As far as the WCA values for TPWF-based blends are concerned, the measured values show an alternating trend with the amount of Co present in the mixture, which can be explained on the basis of two different effects, acting sometimes in opposition: (i) the amount of the most hydrophobic component Co and (ii) the rheological characteristics of the blends since rheology of polymers in the molten state influences the solidification dynamics and therefore the morphology of the films produced by cast extrusion. In fact, as known, film processing affects the morphology of the sample and, consequently, the observed value of contact angle.^46^ Indeed, the surface roughness degree influences the contact angle value according to the characteristics of the asperities in terms of quantity and size.^47^ As a consequence, the measured WCA value is the result of all the abovementioned factors, and if they act in opposition, it will be determined by the prevailing one.

The 70TPWF/30PBS system shows a contact angle of 60°, similar to PBS, which can be explained by taking into account that, at the high temperature of the processing, the PBS present in the blend produces a polymer melt containing well dispersed TPWF starch particles (see Figure 4) with consequent formation, as the sample is cooled down, of a crystalline phase similar to neat PBS and characterized by a smooth surface. The addition of small quantities of copolymer in the formulations 70TPWF/25PBS/5Co and 70TPWF/20PBS/10Co produces a significant increase (values up to 79–80°) of the contact angle, essentially due to both intrinsic higher hydrophobicity of the Co and the increased surface roughness, correlated to the ability of Co to bind with starchy particles of the plasticized flour, causing an increased presence of conglomerates. The increment in roughness caused by starchy conglomerates in these ternary systems is appreciable from SEM micrographs of both the surface (Figure 11, time 0) and cross section of the films (Figure 4). The Wenzel model forecasts indeed that the surface roughness amplifies the wettability of the original surface, with the hydrophilic ones becoming more hydrophilic and the hydrophobic surface becoming more hydrophobic.^48^ It is worthy to mention that the plasticizing action of the copolymer does not correspond to a proper compatibilizer effect due to the low presence of Co in these formulations. When the content of Co is increased to 15 wt % (70TPWF/15PBS/15Co), the compatibilizer effect of the copolymer becomes visible, as evidenced by the reduction in size of starchy conglomerates in the TPWF (see Figure 4). The resulting surface morphology is characterized by rounded asperities, which is determined by a decrease in the contact angle value, which results in close to 72°, despite the increased amount in the blend of the most hydrophobic component (Co). In the 70TPWF/10PBS/20Co blend, the dimensional reduction of the conglomerates and the better dispersion of the starchy particles create a surface with few rounded asperities, to which many small asperities of the “deconglomerated” particles overlap (see Figure 11, time 0); this singular morphology, together with a concomitant increase of the amount of hydrophobic component (Co), causes an increase in the contact angle up to 77°. In the 70TPWF/5PBS/25Co blend, the contact angle drops again to 70° because of the presence of the highest quantity of Co, which, as previously described, favors the dispersion and the plasticization of the starchy particles, with consequent formation of a smooth surface, due to a uniform and almost free section of discrete conglomerates. The relationships highlighted among the contact angle and morphology of surfaces and sections are confirmed by the results of mechanical tensile tests. The ternary systems show a progressive decrease in the Young’s modulus, which corresponds to the increase of strain (at maximum stress) as the quantity of copolymer increases. This trend is in line with the considerations on morphology and with the expected results. The trend of maximum stress, although decreasing as the amount of Co in the mixture increases, shows a relative maximum for 70TPWF/15PBS/20Co, which indeed shows a good dispersion and compatibilization of the small starch particles that work as reinforcement, improving the strength. The excessive plasticization of the same particles for 70TPWF/5PBS/25Co prevents their reinforcement function by lowering the mechanical strength.

## 4 Conclusions

The shift from a linear economy, which produces large quantities of plastic waste with consequent severe environmental problems, to a circular one cannot disregard the replacement of the fossil-based and not biodegradable traditional plastics with bioplastics. To date, the latter is facing serious problems of success in the market, mainly due to their costs being decidedly higher than those of traditional plastics.

The results obtained in this work represent a step forward to the affirmation of bioplastics on the market. We indeed propose a new low-cost bioplastic, prepared through a simple, cost-effective, and eco-friendly technological process. The new material is a ternary blend formed by the cheap plasticized wheat flour, which represents the main component (70 wt %), and by varying amounts of two synthetic polyesters: poly(butylene succinate), which is 100% biobased, compostable, and with mechanical properties similar to LDPE, and a random PBS-based copolymer, containing six-C aliphatic ring moieties, also completely obtainable from renewable resources.

In the ternary blends, the PBS-based copolymer plays an efficient compatibilizer role, as proven by the more than satisfactory final mechanical performances, without compromising the characteristic compostability of wheat flour. In particular, the results obtained evidence that the blends with 15 and 20 wt% copolymers are the best solution, being characterized by a significant improvement in film deformation while keeping good values of both elastic modulus and stress.

Last but not the least, it is interesting to note that, by acting on the PBS/copolymer ratio, it is also possible to modulate the compostability rate of the final material and the mechanical performances.

## References

[ref1] Luo X.; Li J.; Lin X. Effect of Gelatinization and Additives on Morphology and Thermal Behavior of Corn Starch/PVA Blend Films. Carbohydr. Polym. 2012, 90, 1595–1600. 10.1016/j.carbpol.2012.07.036. 22944421

[ref2] Moad G. Chemical Modification of Starch by Reactive Extrusion. Prog. Polym. Sci. 2011, 36, 218–237. 10.1016/j.progpolymsci.2010.11.002.

[ref3] Pereira A. G. B.; Paulino A. T.; Nakamura C. V.; Britta E. A.; Rubira A. F.; Muniz E. C. Effect of Starch Type on Miscibility in Poly(Ethylene Oxide) (PEO)/Starch Blends and Cytotoxicity Assays. Mater. Sci. Eng. C 2011, 31, 443–451. 10.1016/j.msec.2010.11.004.

[ref4] Zullo R.; Iannace S. The Effects of Different Starch Sources and Plasticizers on Film Blowing of Thermoplastic Starch: Correlation among Process, Elongational Properties and Macromolecular Structure. Carbohydr. Polym. 2009, 77, 376–383. 10.1016/j.carbpol.2009.01.007.

[ref5] Yu L.; Dean K.; Li L. Polymer Blends and Composites from Renewable Resources. Prog. Polym. Sci. 2006, 31, 576–602. 10.1016/j.progpolymsci.2006.03.002.

[ref6] Xie F.; Halley P. J.; Avérous L. Rheology to Understand and Optimize Processibility, Structures and Properties of Starch Polymeric Materials. Prog. Polym. Sci. 2012, 37, 595–623. 10.1016/j.progpolymsci.2011.07.002.

[ref7] Pedroso A. G.; Rosa D. S. Mechanical, Thermal and Morphological Characterization of Recycled LDPE/Corn Starch Blends. Carbohydr. Polym. 2005, 59, 1–9. 10.1016/j.carbpol.2004.08.018.

[ref8] Shujun W.; Jiugao Y.; Jinglin Y. Preparation and Characterization of Compatible Thermoplastic Starch/Polyethylene Blends. Polym. Degrad. Stab. 2005, 87, 395–401. 10.1016/j.polymdegradstab.2004.08.012.

[ref9] Ramis X.; Cadenato A.; Salla J. M.; Morancho J. M.; Vallés A.; Contat L.; Ribes A. Thermal Degradation of Polypropylene/Starch-Based Materials with Enhanced Biodegradability. Polym. Degrad. Stab. 2004, 86, 483–491. 10.1016/j.polymdegradstab.2004.05.021.

[ref10] Teyssandier F.; Cassagnau P.; Gérard J. F.; Mignard N.; Mélis F. Morphology and Mechanical Properties of PA12/Plasticized Starch Blends Prepared by High-Shear Extrusion. Mater. Chem. Phys. 2012, 133, 913–923. 10.1016/j.matchemphys.2012.01.117.

[ref11] Teyssandier F.; Cassagnau P.; Gérard J. F.; Mignard N. Reactive Compatibilization of PA12/Plasticized Starch Blends: Towards Improved Mechanical Properties. Eur. Polym. J. 2011, 47, 2361–2371. 10.1016/j.eurpolymj.2011.09.017.

[ref12] Ma X.; Chang P. R.; Yu J.; Wang N. Preparation and Properties of Biodegradable Poly(Propylene Carbonate)/Thermoplastic Dried Starch Composites. Carbohydr. Polym. 2008, 71, 229–234. 10.1016/j.carbpol.2007.05.033.

[ref13] Wang N.; Yu J.; Chang P. R.; Ma X. Influence of Citric Acid on the Properties of Glycerol-Plasticized Dry Starch (DTPS) and DTPS/Poly(Lactic Acid) Blends. Starch/Staerke 2007, 59, 409–417. 10.1002/star.200700617.

[ref14] Ni H. K.; Yang B.; Sun H.; Xu G. Z. Wet Blending of Pregelatinized Starch and Poly(Butylene Succinate). Adv. Mater. Res. 2012, 557-559, 1121–1126. 10.4028/www.scientific.net/AMR.557-559.1121.

[ref15] Lai S. M.; Huang C. K.; Shen H. F. Preparation and Properties of Biodegradable Poly(Butylene Succinate)/Starch Blends. J. Appl. Polym. Sci. 2005, 97, 257–264. 10.1002/app.21679.

[ref16] Wang W.; Zhang G.; Zhang W.; Guo W.; Wang J. Processing and Thermal Behaviors of Poly (Butylene Succinate) Blends with Highly-Filled Starch and Glycerol. J. Polym. Environ 2013, 21, 46–53. 10.1007/s10924-012-0505-7.

[ref17] Zeng J. B.; Jiao L.; Li Y. D.; Srinivasan M.; Li T.; Wang Y. Z. Bio-Based Blends of Starch and Poly(Butylene Succinate) with Improved Miscibility, Mechanical Properties, and Reduced Water Absorption. Carbohydr. Polym. 2011, 83, 762–768. 10.1016/j.carbpol.2010.08.051.

[ref18] Suchao-In K.; Koombhongse P.; Chirachanchai S. Starch Grafted Poly(Butylene Succinate) via Conjugating Reaction and Its Role on Enhancing the Compatibility. Carbohydr. Polym. 2014, 102, 95–102. 10.1016/j.carbpol.2013.11.001. 24507260

[ref19] Yin Q.; Chen F.; Zhang H.; Liu C. Fabrication and Characterisation of Thermoplastic Starch/Poly(Butylene Succinate) Blends with Maleated Poly(Butylene Succinate) as Compatibiliser. Plast., Rubber Compos. 2015, 44, 362–367. 10.1179/1743289815Y.0000000031.

[ref20] Suttiruengwong S.; Sotho K.; Seadan M. Effect of Glycerol and Reactive Compatibilizers on Poly(Butylene Succinate)/Starch Blends. J. Renew. Mater. 2014, 2, 85–92. 10.7569/JRM.2013.634135.

[ref21] Tynski P.; Ostrowska J.; Paluch M.; Sadurski W. Properties of Biodegradable Films Based on Thermoplastic Starch and Poly(Butylene Succinate) with Plant Oil Additives. In Proceedings of the 2nd International Scientific Conference ≪Chemical Technology and Engineering≫; Lviv Polytechnic National University, 2019; pp 257–261.

[ref22] Zhang S.; He Y.; Yin Y.; Jiang G. Fabrication of Innovative Thermoplastic Starch Bio-Elastomer to Achieve High Toughness Poly(Butylene Succinate) Composites. Carbohydr. Polym. 2019, 206, 827–836. 10.1016/j.carbpol.2018.11.036. 30553390

[ref23] Kim H. S.; Kim H. J.; Lee J. W.; Choi I. G. Biodegradability of Bio-Flour Filled Biodegradable Poly(Butylene Succinate) Bio-Composites in Natural and Compost Soil. Polym. Degrad. Stab. 2006, 91, 1117–1127. 10.1016/j.polymdegradstab.2005.07.002.

[ref24] Ohkita T.; Lee S.-H. Effect of Aliphatic Isocyanates (HDI and LDI) as Coupling Agents on the Properties of Eco-Composites from Biodegradable Polymers and Corn Starch. J. Adhes. Sci. Technol. 2004, 18, 905–924. 10.1163/156856104840516.

[ref25] Ayu R. S.; Khalina A.; Harmaen A. S.; Zaman K.; Jawaid M.; Lee C. H. Effect of Modified Tapioca Starch on Mechanical, Thermal, and Morphological Properties of PBS Blends for Food Packaging. Polymers 2018, 10, 1187 10.3390/polym10111187. PMC6290640 30961112

[ref26] Li J.; Luo X.; Lin X.; Zhou Y. Comparative Study on the Blends of PBS/Thermoplastic Starch Prepared from Waxy and Normal Corn Starches. Starch/Staerke 2013, 65, 831–839. 10.1002/star.201200260.

[ref27] Ku-marsilla K. I.; Verbeek C. J. R. Compatibilization of Protein Thermoplastics and Polybutylene Succinate Blends. Macromol. Mater. Eng. 2015, 300, 161–171. 10.1002/mame.201400141.

[ref28] Quattrosoldi S.; Soccio M.; Gazzano M.; Lotti N.; Munari A. Fully Biobased, Elastomeric and Compostable Random Copolyesters of Poly(Butylene Succinate) Containing Pripol 1009 Moieties: Structure-Property Relationship. Pol Deg Stab 2020, 178, 109189 10.1016/j.polymdegradstab.2020.109189.

[ref29] Puglia D.; Dominici F.; Kenny J. M.; Santulli C.; Governatori C.; Tosti G.; Benincasa P. Tensile Behavior of Thermoplastic Films from Wheat Flours as Function of Raw Material Baking Properties. J. Polym. Environ. 2016, 24, 37–47. 10.1007/s10924-015-0745-4.

[ref30] Benincasa P.; Dominici F.; Bocci L.; Governatori C.; Panfili I.; Tosti G.; Torre L.; Puglia D. Relationships between Wheat Flour Baking Properties and Tensile Characteristics of Derived Thermoplastic Films. Ind. Crops Prod. 2017, 100, 138–145. 10.1016/j.indcrop.2017.02.021.

[ref31] Chen M. S.; Chang S. J.; Chang R. S.; Kuo W. F.; Tsai H. B. Copolyesters. I. Sequence Distribution of Poly(Butylene Terephthalate-Co-Adipate) Copolyesters Determined by 400 MHz NMR. J . Appl. Polym. 1990, 40, 1053–1057. 10.1002/app.1990.070400537.

[ref32] Soccio M.; Lotti N.; Gazzano M.; Govoni M.; Giordano E.; Munari A. Molecular Architecture and Solid-State Properties of Novel Biocompatible PBS-Based Copolyesters Containing Sulphur Atoms. React. Funct. Polym. 2012, 72, 856–867. 10.1016/j.reactfunctpolym.2012.08.002.

[ref33] Chrissafis K.; Paraskevopoulos K. M.; Bikiaris D. N. Thermal Degradation Mechanism of Poly(Ethylene Succinate) and Poly(Butylene Succinate): Comparative Study. Thermochim. Acta 2005, 435, 142–150. 10.1016/j.tca.2005.05.011.

[ref34] Gan Z.; Abe H.; Kurokawa H.; Doi Y. Solid-State Microstructures, Thermal Properties, and Crystallization of Biodegradable Poly(Butylene Succinate) (PBS) and Its Copolyesters. Biomacromolecules 2001, 2, 605–613. 10.1021/bm015535e. 11749227

[ref35] Chabrat E.; Abdillahi H.; Rouilly A.; Rigal L. Influence of Citric Acid and Water on Thermoplastic Wheat Flour/Poly(Lactic Acid) Blends. I: Thermal, Mechanical and Morphological Properties. Ind. Crops Prod. 2012, 37, 238–246. 10.1016/j.indcrop.2011.11.034.

[ref36] Abdillahi H.; Chabrat E.; Rouilly A.; Rigal L. Influence of Citric Acid on Thermoplastic Wheat Flour/Poly(Lactic Acid) Blends. II. Barrier Properties and Water Vapor Sorption Isotherms. Ind. Crops Prod. 2013, 50, 104–111. 10.1016/j.indcrop.2013.06.028.

[ref37] Huang Z.; Qian L.; Yin Q.; Yu N.; Liu T.; Tian D. Biodegradability Studies of Poly(Butylene Succinate) Composites Filled with Sugarcane Rind Fiber. Polym. Test. 2018, 66, 319–326. 10.1016/j.polymertesting.2018.02.003.

[ref38] Thellen C.; Orroth C.; Froio D.; Ziegler D.; Lucciarini J.; Farrell R.; D’Souza N. A.; Ratto J. A. Influence of Montmorillonite Layered Silicate on Plasticized Poly(l-Lactide) Blown Films. Polymer 2005, 46, 11716–11727. 10.1016/j.polymer.2005.09.057.

[ref39] Sreekumar P. A.; Leblanc N.; Saiter J. M. Effect of Glycerol on the Properties of 100% Biodegradable Thermoplastic Based on Wheat Flour. J. Polym. Environ 2013, 21, 388–394. 10.1007/s10924-012-0497-3.

[ref40] Garalde R. A.; Thipmanee R.; Jariyasakoolroj P.; Sane A. The Effects of Blend Ratio and Storage Time on Thermoplastic Starch/Poly(Butylene Adipate-Co-Terephthalate) Films. Heliyon 2019, 5, e01251 10.1016/j.heliyon.2019.e01251. 31016252 PMC6475639

[ref41] Imre B.; García L.; Puglia D.; Vilaplana F. Reactive Compatibilization of Plant Polysaccharides and Biobased Polymers: Review on Current Strategies, Expectations and Reality. Carbohydr. Polym. 2019, 209, 20–37. 10.1016/j.carbpol.2018.12.082. 30732800

[ref42] Bai Z.; Liu Y.; Su T.; Wang Z. Effect of Hydroxyl Monomers on the Enzymatic Degradation of Poly(Ethylene Succinate), Poly(Butylene Succinate), and Poly(Hexylene Succinate). Polymers 2018, 10, 90 10.3390/polym10010090. PMC6414858 30966127

[ref43] Bulatović V. O.; Grgić D. K.; Slouf M.; et al. Biodegradability of Blends Based on Aliphatic Polyester and Thermoplastic Starch. Chem. Pap. 2019, 73, 1121–1134. 10.1007/s11696-018-0663-8.

[ref44] Averous L.; Fauconnier N.; Moro L.; Fringant C. Blends of Thermoplastic Starch and Polyesteramide: Processing and Properties. J. Appl. Polym. Sci. 2000, 76, 1117–1128. 10.1002/(SICI)1097-4628(20000516)76:7<1117::AID-APP16>3.0.CO;2-W.

[ref45] Mahieu A.; Terrié C.; Agoulon A.; Leblanc N.; Youssef B. Thermoplastic Starch and Poly(ε-Caprolactone) Blends: Morphology and Mechanical Properties as a Function of Relative Humidity. J. Polym. Res. 2013, 20, 229 10.1007/s10965-013-0229-y.

[ref46] Garrido T.; Etxabide A.; Peñalba M.; De La Caba K.; Guerrero P. Preparation and Characterization of Soy Protein Thin Films: Processing-Properties Correlation. Mater. Lett. 2013, 105, 110–112. 10.1016/j.matlet.2013.04.083.

[ref47] Lin F. Y. H.; Li D.; Neumann A. W. Effect of Surface Roughness on the Dependence of Contact Angles on Drop Size. J. Colloid Interface Sci. 1993, 159, 86–95. 10.1006/jcis.1993.1300.

[ref48] Seo K.; Kim M.; Kim D. H. Re-Derivation of Young’s Equation, Wenzel Equation, and Cassie-Baxter Equation Based on Energy Minimization. In Surface Energy; Aliofkhazraei M., Ed.; 2015, 10.5772/61066.

